# Racial disparities in outcomes for high‐grade uterine cancer: A California cancer registry study

**DOI:** 10.1002/cam4.1742

**Published:** 2018-08-19

**Authors:** Mana Baskovic, Daphne Y. Lichtensztajn, Trung Nguyen, Amer Karam, Diana P. English

**Affiliations:** ^1^ Department of Obstetrics & Gynecology Stanford University Hospital Stanford California; ^2^ Greater Bay Area Cancer Registry Cancer Prevention Institute of California Fremont California

**Keywords:** California cancer registry, health disparities in African‐American populations, high‐grade uterine cancers, molecular testing in high‐grade uterine cancer, survival disparity by socioeconomic differences

## Abstract

**Background:**

Endometrial cancer (EC) is the most common gynecologic malignancy. We examined factors affecting overall prognosis and survival among different racial groups diagnosed with high‐grade EC.

**Methods:**

We utilized the California Cancer Registry database (CCR) to identify women with high‐grade II EC from 1998 to 2009. Using the Kaplan‐Meier method, we described disease‐specific survival. Survival by stage, race, and time to treatment category was compared using the log‐rank test. The associations of race with disease‐specific survival were modeled using Cox proportional hazards regression. Covariates were selected a priori.

**Results:**

A total of 10 647 patients met study eligibility criteria. The majority of patients in this cohort of high‐grade EC were non‐Hispanic (NH) white (64.1%), followed by Hispanic (15.7%), Asian (10.4%), and NH black (9.8%). NH black women had higher incidence of certain aggressive histologic subtypes in comparison with NH whites, including serous carcinomas and carcinosarcoma.

Non‐Hispanic black patients had a worse 5‐year disease‐specific survival (DSS) when compared to other racial groups. The five‐year DSS for NH black women was 54% (51%‐57%), compared to NH white women 66% (65%‐67%), Hispanic 67% (64%‐69%), and Asians 69% (67%‐72%) (*P* < 0.0001). This clear survival disadvantage of NH black women persisted when controlling for other factors.

**Conclusions:**

Non‐Hispanic black women have a higher incidence of more aggressive histologic subtypes even among a cohort of women high‐grade EC and have a disproportionately worse disease‐specific survival after controlling for factors such as age, histologic subtype, stage, time to treatment, and type of treatment.

## 1 INTRODUCTION

Endometrial cancer is the most commonly diagnosed gynecologic cancer in the United States with an estimated 61 380 new cases in 2017. The majority of endometrial cancers are type I cancers. Type I tumors are often diagnosed at an early stage and tend to have an overall favorable prognosis. However, a proportion of women will be diagnosed with a more aggressive histologic subtype referred to as type II uterine cancer. This historical classification is useful but there is heterogeneity and overlap between the groups. In particular when considering grade 3 endometrioid tumors, there is a challenge with pathologic accuracy due to poor diagnostic reproducibility.^1^ Type II or high‐grade uterine cancer affects approximately 20%‐30% of women with endometrial cancer and is associated with a far greater risk of recurrence and an increased overall mortality.^1^ It is estimated that 10 920 deaths will be attributed to endometrial cancer in 2017, with the majority of these deaths due to the aggressive high‐grade uterine cancers.^2^

The diagnosis of endometrial cancer in the black population can carry a substantially higher mortality in comparison with their white, Hispanic, and Asian counterparts. The age‐adjusted incidence of endometrial cancer has been reported to be 31% lower among black patients compared to white women; however, the age‐adjusted mortality among black women is approximately 84% higher.^3^

Many of the prior studies on disparity in endometrial cancer outcomes including those that have analyzed genetic and molecular tumor features are limited and omit additional confounding variables such as socioeconomic factors, time interval from diagnosis to treatment, type of intervention, and comorbid conditions. We sought to examine factors contributing to inequality in outcomes among racial groups diagnosed with high‐grade uterine cancers by analyzing data from the California Cancer Registry (CCR).

## 2 METHODS

Data were obtained from the California Cancer Registry (CCR). The CCR is a state‐mandated registry that has collected data on all cancers diagnosed in residents of the state since 1988. The CCR is comprised of three regional registries that are members of the National Cancer Institute's (NCI) Surveillance Epidemiology and End Results (SEER) program. The CCR extracts information on patient demographics, tumor characteristics, and treatment. Vital status information is routinely updated through regular linkages with state and national databases as well as active hospital follow‐up.

### 2.1 Study population and eligibility

**Figure 1:**
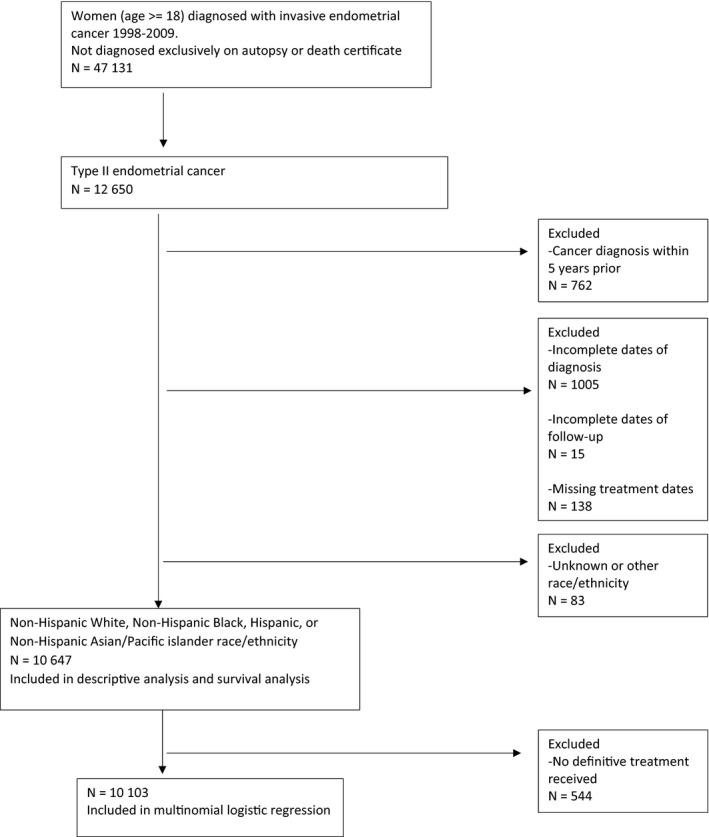
Graphic of cohort selection diagram

We obtained data on all women (age ≥18) diagnosed with invasive endometrial cancers from 1998 to 2009, excluding those diagnosed by death certificate or autopsy only (N = 47 131; Figure 1). We further restricted our selection to high‐grade endometrial cancers based on International Classification of Diseases for Oncology, Third edition (ICD‐O‐3) morphology codes, detailed below (N = 12 650).

Patients with a prior cancer diagnosis within 5 years of the tumor of interest (N = 762), those with incomplete dates of diagnosis (N = 1005) or incomplete follow‐up (N = 15), or with missing treatment dates (N = 138) were excluded. The cohort was further limited to non‐Hispanic white, non‐Hispanic black, Hispanic, and non‐Hispanic Asian/Pacific Islanders (PI).

The final cohort included 10 647 women. Histologic subtypes were categorized using ICD‐O‐3 codes as follows: serous carcinoma (8441, 8460, 8461); clear cell carcinoma (8310, 8005); carcinosarcoma/malignant mixed Mullerian tumor (8950, 8951, 8980, 8981), high‐grade endometrioid (8140, 8210, 8211, 8260, 8261, 8262, 8263, 8340, 8380, 8381, 8382, 8383, 8384, 8560, 8570, restricted to cases with grade coded as high)^4^; and mixed (8323, 8255) high‐grade uterine cancer.

### 2.2 Measures

SEER does not extract International Federation of Gynecology and Obstetrics (FIGO) stage directly. We derived the FIGO stage using data on tumor extension, lymph node involvement, and presence of metastases. The CCR does not collect data on individual‐level measures of socioeconomic status (SES). A previously described composite measure was used to assign each case to a quintile of neighborhood SES (nSES) based on the Census block group of the geocoded address at the time of diagnosis.^5^, ^6^

A Charlson Index derived from linkage of CCR data with statewide hospital discharge data was available from CCR and was used as a measure comorbidity burden.^7^ Scores were categorized as 0 (no comorbidity), 1, and 2 or higher.

Time to treatment was calculated in days from the date of diagnosis to the date of definitive surgery (hysterectomy), radiation, or chemotherapy, which ever was the earliest. Time to treatment was categorized as ≤2 weeks, 2‐4 weeks, and >4 weeks.

### 2.3 Statistical methods

The association of patient and tumor characteristics with time to initiation of treatment was modeled using multinomial logistic regression. Variables included in the model were selected a priori and included race/ethnicity, age, stage, histologic subtype, type of treatment first received, insurance status, nSES, marital status, NCI cancer center status, and year of diagnosis. Two to four weeks to treatment was used as the referent category.

Disease‐specific survival analysis was conducted. To ensure a minimum five‐year potential follow‐up for the survival analysis, only cases diagnosed between 1998 and 2009 were included (N = 10 647). Survival time was calculated in days from date of diagnosis to date of death or last contact. Vital status data were complete through 31 December 2014. Any patient alive at this date was censored at this time. For disease‐specific survival analysis, patients who died from a cause other than uterine cancer were censored on the date of death. Using the Kaplan‐Meier method, we described disease‐specific survival. Survival by stage, race/ethnicity, and time to treatment category was compared using the log‐rank test. The associations of race/ethnicity with disease‐specific survival was modeled using Cox proportional hazards regression. Covariates were selected a priori and included age, histologic subtype, stage, time to treatment, type of treatment, nSES, NCI cancer center, and year of diagnosis. Proportional hazards assumptions were tested using Schoenfeld residuals and log‐log plots.

Statistical analyses were performed using SAS version 9.4 (SAS Institute, Cary, NC). Tests were two‐sided, with *P* < 0.05 considered statistically significant.

## 3 RESULTS

Our study included 10 647 patients who met the eligibility criteria for survival analysis. Median follow‐up was 9.1 years for nondeceased patients (interquartile range: 6.8‐12.3 years).

### 3.1 Characteristics of entire cohort

The majority of patients in this cohort of high‐grade endometrial cancer were non‐Hispanic (NH) white (64.1%), followed by Hispanic (15.7%), Asian (10.4%), and NH black (9.8%). Most patients were diagnosed between the age of 50 and 79 (75.6%), with 8.4% <50, and 16.0% eighty or older. High‐grade endometrioid adenocarcinoma was the most common histologic subtype (43.9%), followed by serous (17.6%), carcinosarcoma (17.2%), mixed (16.0%), and clear cell (5.2%). Most patients were diagnosed at stage I (44.4%), followed by stage III (23.5%), stage IV (19.0%), stage II (9.3%), and unknown (3.9%). About 36.1% of patients received radiation, while 27.8% received chemotherapy. Most patients were reasonably healthy with Charlson Comorbidity Index (CCI) of 0 (62.6%). Approximately 14.2% of patients were in the lowest quintile of neighborhood socioeconomic status (nSES), and 23.8% were among highest SES. Approximately half of the patients had private insurance (51.2%), followed by Medicare (31.1%), and Public/Medicaid, VA (12.1%).

### 3.2 Comparisons of patient characteristics and outcomes by race/ethnicity

NH black women had higher incidence of certain aggressive histologic subtypes in comparison with NH whites, including serous carcinoma (24.4% vs 16.3%, *P* < 0.0001) and carcinosarcoma (24.9% vs 16.0%, *P* < 0.0001; Table 1). NH white patients were more likely to have stage I disease compared to NH black women (45.8% vs 34.9%, *P* = 0.0001). NH black women were less likely to have a lymph node dissection compared to NH white patients (*P* < 0.0001). Also among patients who had lymph node dissections, NH white patients were more likely to have 10 or greater lymph nodes removed compared to NH black patients (*P* = 0.0044). NH black women were more likely to reside in the lowest nSES (30.2) %, compared to 8.4% of NH white women (*P* < 0.0001; Table 1).

**Table 1:** Characteristics of women diagnosed with high‐grade endometrial cancer, CCR 1998‐2009 by race/ethnicity

	NH White		NH Black		Hispanic		Asian/PI		Chi‐square *P* value
	N	%	N	%	N	%	N	%	
Age									
<50	431	6.3	56	5.4	261	15.6	150	13.5	<0.0001
50‐59	1355	19.9	196	18.8	417	24.9	343	30.9	
60‐69	1850	27.1	412	39.5	517	30.9	314	28.3	
70‐79	1836	26.9	268	25.7	331	19.8	206	18.5	
80+	1348	19.8	111	10.6	147	8.8	98	8.8	
Histologic subtype									
Serous	1113	16.3	254	24.4	316	18.9	188	16.9	<0.0001
High‐grade endometrioid	3141	46.1	331	31.7	699	41.8	508	45.7	
Clear cell	344	5.0	61	5.8	90	5.4	63	5.7	
Carcinosarcoma/Mullerian mixed	1094	16.0	260	24.9	304	18.2	173	15.6	
Mixed	1128	16.5	137	13.1	264	15.8	179	16.1	
FIGO stage equivalent									
I	3126	45.8	411	39.4	705	42.1	484	43.6	<0.0001
II	614	9.0	111	10.6	162	9.7	99	8.9	
III	1574	23.1	244	23.4	394	23.6	290	26.1	
IV	1241	18.2	217	20.8	347	20.7	215	19.4	
Unknown	265	3.9	60	5.8	65	3.9	23	2.1	
Number LN examined									
None	2061	30.2	423	40.6	585	35.0	282	25.4	<0.0001
1‐9	1432	21.0	223	21.4	314	18.8	244	22.0	
10 or more	3059	44.9	368	35.3	731	43.7	555	50.0	
Unknown	268	3.9	29	2.8	43	2.6	30	2.7	
Number LN positive^a^									
None	3528	74.1	424	68.4	785	72.2	592	71.4	0.0820
1	379	8.0	62	10.0	105	9.7	85	10.3	
2‐5	528	11.1	77	12.4	122	11.2	90	10.9	
>5	255	5.4	49	7.9	64	5.9	52	6.3	
Unknown	69	1.4	8	1.3	12	1.1	10	1.2	
Radiation									
None	4379	64.2	711	68.2	1037	62.0	679	61.1	0.0241
External Beam	1509	22.1	206	19.8	406	24.3	295	26.6	
Brachytherapy	330	4.8	44	4.2	77	4.6	39	3.5	
Beam + Brachytherapy	542	7.9	72	6.9	142	8.5	91	8.2	
Radiation, NOS	59	0.9	10	1.0	11	0.7	7	0.6	
Unknown	<5	0.0	0	0	0	0	0	0	
Chemotherapy									
No	5041	73.9	759	72.8	1128	67.4	756	68.0	<0.0001
Yes	1779	26.1	284	27.2	545	32.6	355	32.0	
Charlson Comorbidity Index									
Unknown	318	4.7	64	6.1	172	10.3	87	7.8	<0.0001
0	4500	66.0	535	51.3	936	55.9	697	62.7	
1	1379	20.2	255	24.4	388	23.2	250	22.5	
2+	623	9.1	189	18.1	177	10.6	77	6.9	
Neighborhood SES quintile									
1‐ Lowest SES	573	8.4	315	30.2	515	30.8	108	9.7	<0.0001
2	1104	16.2	279	26.7	438	26.2	192	17.3	
3 ‐ Middle SES	1521	22.3	212	20.3	326	19.5	232	20.9	
4	1660	24.3	148	14.2	240	14.3	284	25.6	
5 ‐ Highest SES	1962	28.8	89	8.5	154	9.2	295	26.6	
Primary payer									
None	87	1.3	25	2.4	97	5.8	36	3.2	<0.0001
Private	3548	52.0	520	49.9	778	46.5	605	54.5	
Public/Medicaid, VA	489	7.2	178	17.1	396	23.7	221	19.9	
Medicare	2469	36.2	302	29.0	333	19.9	205	18.5	
Unknown	227	3.3	18	1.7	69	4.1	44	4.0	
Seen at NCI‐designated cancer center									
No	6069	89.0	913	87.5	1367	81.7	913	82.2	<0.0001
Yes	751	11.0	130	12.5	306	18.3	198	17.8	
Total	6820	100.0	1043	100.0	1673	100.0	1111	100.0	

a Among women with lymph nodes removed.

In the multivariable model, compared to NH white, patients who are Hispanic and NH black were more likely to receive care greater than 4 weeks vs within 2‐4 weeks from diagnosis (Odds ratio (OR) 1.28, CI:1.10‐1.49, OR 1.40, CI: 1.17‐1.68, respectively). There was an association of increasing age with receipt of treatment later than 4 weeks (OR 1.01, CI: 1.00‐1.01, per year of age). Women with CCI score of 1 or 2 were more likely to receive later treatment than women with no significant health issue (OR 1.31, CI: 1.15‐1.48, OR 1.23, CI: 1.03‐1.47, respectively). NH black patients were more likely to have a CCI score of 2 or greater compared to their counterparts in the other racial groups (Table 1).

Women from neighborhoods in the lowest quintile of SES were almost twice as likely to receive later treatment than those from the highest quintile of neighborhood SES (OR 1.93, CI: 1.61‐2.31). Compared to women with private insurance, those with no insurance and those with Medicaid or VA were almost twice as likely to be treated later than 4 weeks from diagnosis vs 2‐4 weeks (OR 1.94, CI: 1.30‐2.89 and OR 1.74, CI: 1.45‐2.09), respectively) (data not shown).

**Figure 2:**
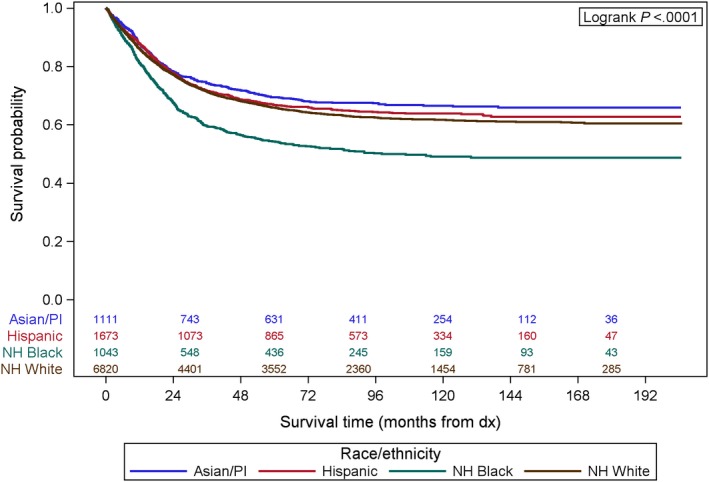
Kaplan‐Meier curve showing 5‐y disease‐specific survival by race/ethnicity

Non‐Hispanic black patients had a worse 5‐year disease‐specific survival (DSS) when compared to other racial/ethnic groups (Figure 2). The five‐year DSS for NH black women was 54% (51‐57%), compared to NH white women 66% (65‐67%), Hispanic 67% (64‐69%) and Asians 69% (67‐72%) (*P* < 0.0001).

### 3.3 The effect of time to treatment on disease‐specific survival by race/ethnicity and by histologic subtype

**Figure 3:**
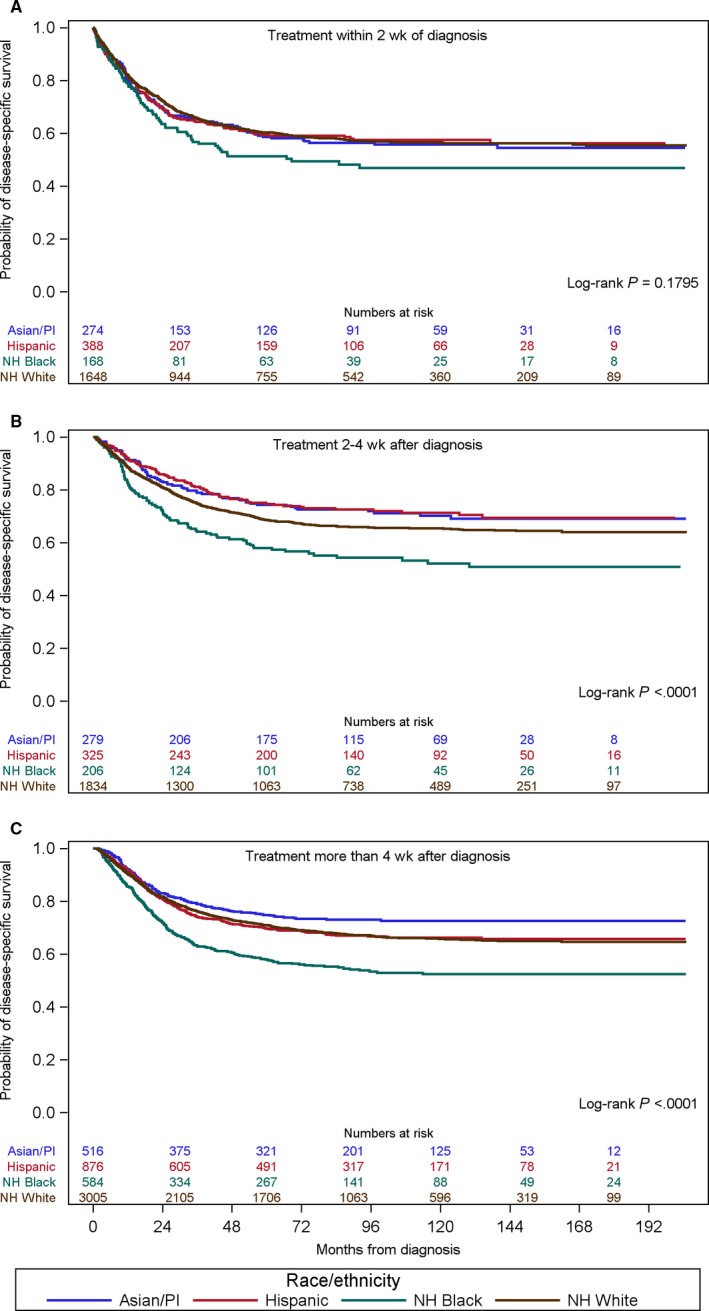
Kaplan‐Meier curve showing disease‐specific survival by race/ethnicity within the time to treatment categories. A, Treatment within 2 wk of diagnosis. B, Treatment 2‐4 wk after diagnosis. C, Treatment more than 4 wk after diagnosis

When DSS was stratified by race within time to treatment, NH black patients did not have a worse DSS when treated within 2 weeks from diagnosis, based on Kaplan‐Meier analysis (*P* = 0.1795; Figure 3).

However, when time to treatment was between 2 and 4 weeks or greater than 4 weeks, NH black patients had a significantly worse DSS compared to patients of other race/ethnicity (*P* < 0.0001, *P* < 0.0001, respectively; Figure 3). All women regardless of race, treated at less than or equal to 2 weeks’ time from diagnosis to intervention had a poorer outcome compared to their counterparts who received treatment between 2 and 4 weeks or greater than 4 weeks from diagnosis (data not shown). Patients treated in the ≤2 weeks interval were more likely to have a diagnosis of carcinosarcoma compared to high‐grade endometrioid (HR 1.41, 95% CI 1.24‐1.60). Patients treated in this time frame were also more likely to be stage III compared to stage I and more likely to be stage IV vs stage I, (HR 1.18, 95% CI 1.05‐1.34, and HR 2.67, 95% CI 2.35‐3.03, respectively) (data not shown).

### 3.4 Multivariable model of associations with disease‐specific survival

Using Cox proportional hazard multivariable analysis, after adjusting for age, histologic subtype, type of treatment, time to treatment, comorbidity, insurance status, socioeconomic status, marital status, NCI cancer center, and year of diagnosis, NH black women still had an increased hazard of disease‐specific death compared to NH white women (HR 1.21, 95% CI 1.09‐1.35; Table 2).

**Table 2:** Associations with disease‐specific survival (hazard ratios and 95% confidence intervals)

		Hazard ratio	95% confidence interval	*P* value
Race/ethnicity	Asian/PI vs NH White	0.95	0.84‐1.06	0.3476
	Hispanic vs NH White	0.94	0.85‐1.04	0.2083
	**NH Black vs NH White**	**1.21**	**1.09‐1.35**	**0.0004**
Age	**Per year**	**1.02**	**1.02‐1.02**	**<0.0001**
FIGO stage	**II vs I**	**2.21**	**1.92‐2.54**	**<0.0001**
	**III vs I**	**4.36**	**3.94‐4.82**	**<0.0001**
	**IV vs I**	**10.39**	**9.35‐11.55**	**<0.0001**
	**Unknown vs I**	**2.21**	**1.83‐2.67**	**<0.0001**
Histologic subtype	**Carcinosarcoma vs High‐grade endometrioid**	**1.88**	**1.72‐2.04**	**<0.0001**
	Clear cell vs High‐grade endometrioid	0.99	0.85‐1.16	0.9180
	**Mixed vs High‐grade endometrioid**	**0.68**	**0.61‐0.77**	**<0.0001**
	Serous vs High‐grade endometrioid	1.09	0.99‐1.19	0.0745
Hysterectomy	**No vs Yes**	**1.99**	**1.74‐2.27**	**<0.0001**
Radiation	No vs Yes	1.04	0.97‐1.12	0.2565
Chemotherapy	No vs Yes	1.08	1.00‐1.17	0.0625
Charlson comorbidity score	**1 vs 0**	**1.19**	**1.10‐1.30**	**<0.0001**
	**2+ vs 0**	**1.44**	**1.30‐1.61**	**<0.0001**
	Unknown vs 0	0.97	0.84‐1.11	0.6388
Time to treatment	**≤2 weeks vs 2‐4 weeks**	**1.14**	**1.03‐1.25**	**0.0076**
	>4 weeks vs 2‐4 weeks	0.93	0.86‐1.02	0.1233
	**No treatment vs 2‐4 weeks**	**2.07**	**1.71‐2.52**	**<0.0001**
Primary payer	**Medicare vs Private**	**1.09**	**1.01‐1.18**	**0.0304**
	None vs Private	0.99	0.77‐1.27	0.9353
	Public/Medicaid, VA vs Private	1.06	0.95‐1.19	0.2705
	Unknown vs Private	0.99	0.81‐1.20	0.9053
Neighborhood SES	1 vs 5 (highest)	1.08	0.96‐1.22	0.1808
	2 vs 5 (highest)	1.06	0.96‐1.18	0.2417
	**3 vs 5 (highest)**	**1.11**	**1.01‐1.23**	**0.0323**
	4 vs 5 (highest)	1.00	0.91‐1.11	0.9389
Marital status	**Not married vs Married**	**1.10**	**1.02‐1.18**	**0.0084**
	Unknown vs Married	0.93	0.75‐1.15	0.4845
NCI cancer center	**Yes vs No**	**0.88**	**0.79‐0.97**	**0.0110**
Year of diagnosis	Per year	0.99	0.98‐1.00	0.2673

Statistically significant values are in bold.

In the multivariate analysis, stage at diagnosis had the greatest effect on disease‐specific survival. Women diagnosed with stage IV disease had a ten‐fold greater risk of disease‐specific mortality compared to those diagnosed at stage I (HR 10.39, CI: 9.35‐11.55). Age appeared to play a significant role with a 2% increased risk of death of disease per year of age. Patients with carcinosarcoma had an increased risk of disease‐specific death compared to patients with high‐grade endometrioid carcinoma (HR 1.88, CI: 1.74‐2.27), whereas those with mixed histology had a decreased hazard of disease‐specific mortality compared to those with high‐grade endometroid histology (HR 0.68, CI: 0.61‐0.77). Not surprisingly, patients who did not have a hysterectomy were much more likely to die of disease (HR 1.99, CI: 1.74‐2.27).

Patients who were treated within the first 2 weeks after diagnosis had an elevated risk of disease‐specific death compared to those treated between 2 and 4 weeks after diagnosis (HR 1.14, CI: 1.03‐1.25), but interestingly there was no significant association with disease‐specific mortality for greater than 4 weeks from diagnosis to treatment. Notably, patients with a diagnosis of carcinosarcoma had a worse 5‐yr DSS within each time to treatment category (≤2 weeks, 2‐4 weeks, and > 4 weeks).

Patients with more comorbid conditions (CCI 1 or 2 + ) were more likely to die of disease compared to their peers with CCI 0. Compared to patients with private insurance, those with Medicare had an elevated risk of disease‐specific death (HR 1.09, CI: 1.01‐1.18). Treatment at a NCI cancer center was associated with lower risk of disease‐specific death (HR 0.88, CI 0.79‐0.97; Table 2).

We also evaluated the association of race/ethnicity with disease‐specific survival by histologic subtype in multivariable model stratified by stage and adjusted for age, treatment (hysterectomy, chemotherapy, radiation), time to treatment, Charlson comorbidity score, primary payer, neighborhood SES, marital status, care at NCI‐designated cancer center, and year of diagnosis. NH black patients compared to NH white patients have an increased hazard for death even within some of the less favorable subtypes. Specifically, the elevated hazard ratios were found for patients with carcinosarcoma, serous, and mixed subtypes. There was no significant difference for clear cell or high‐grade endometrioid histology (data not shown).

## 4 DISCUSSION

Although Latinos and African Americans comprise approximately 30% of the population in the USA, these patients account for just about 6% of those enrolled in federally funded clinical trials.^8^ Racial/ethnic minority groups including Native Hawaiians, Asian Americans, American Indians, and PI were found to be significantly underrepresented in cancer clinical trials. Also less than 2% of these government‐funded cancer research studies looked at minority health needs.^9^ This in itself may explain the worse outcomes for minority groups such as NH black patients when treated using the same treatment paradigms recommended based on superiority in clinical trials; however, these trials likely include populations that may have been largely socially and genetically different.

Racial disparities in care and outcomes are pervasive in medicine and not only in the field of oncology. Race differences also exist in the performance of potential life‐saving interventions, such as invasive cardiac procedures.^10^ There is no doubt that the reasons behind these observations are complex. In our review of high‐grade uterine cancers in the CCR, the mortality of high‐grade endometrial cancer varies greatly across all racial/ethnic groups. Specifically, NH black women have an overall poorer prognosis compared to all other racial groups. This extreme differentiation in an already poor prognosis group of endometrial cancer patients is multifactorial. Factors that may serve to contribute to such differences include molecular/genetic differences, socioeconomic, access to specialized centers, time to treatment, and type of treatment received.

The survival disparity is highlighted when evaluating the socioeconomic differences across the various racial groups. In our database study, NH black women were more likely to reside in the lowest nSES 30.5%, compared to 8.3% of NH white women and were more likely to be unmarried and enrolled in a government‐sponsored insurance program compared NH white patients. Lower socioeconomic status and lack of healthcare funding may explain some of the inequalities in definitive treatment for this patient population. Compared to NH white, patients who are Hispanic and NH black were more likely to receive care greater than 4 weeks vs within 2‐4 weeks from diagnosis although this treatment delay did not significantly affect outcome in this study of high‐risk endometrial cancer. NH black and Hispanic patients may have delays in receiving treatment due to lack of access to appropriate tertiary care centers, long waiting periods for enrollment into government‐sponsored programs and further delay with awaiting appointment scheduling. These effects are often difficult to quantify given that individual factors cannot be appropriately teased out from a large database study. These data may become more important when assessing factors affecting outcomes in less aggressive conditions than high‐grade uterine cancers.

Non‐Hispanic black patients compared to NH white patients accounted for 9.8% vs 64.1% of this study population, respectively, even though according to the 2015 United States Census Bureau estimates, California's population was 6.5% black and 72.9% white. In general, NH black women present more commonly with aggressive subtypes of endometrial cancer and at a more advanced stage. In our study, the incidence of all aggressive histologic subtypes of high‐grade endometrial cancers remained higher in the NH black population as well: serous (23.8% vs 15.4%), clear cell (5.9% vs 4.8%), and carcinosarcoma (24.5% vs 15.1%).

Molecular differences between endometrial cancers in NH black and NH white women has been studied previously and included identifying differences in p53 mutations, Her2/neu expression, and PTEN mutations. Mutations in tumor suppressor gene p53 are associated with overall poorer prognosis. Alkushi et al^11^ reported higher p53 mutation expression in tumors of African American women. Similarly, HER2/neu oncogene expression has been associated with treatment resistance and as a result, poor survival. A study by Maxwell et al^12^ in 2007 found heavy Her2/neu receptor expression in African American women with papillary serous carcinoma (70%) vs white women (24%). Against this background, the molecular profiles of endometrial cancer within racial groups were evaluated in The Cancer Genome Atlas (TCGA). Black patients were more likely to have copy number variant (CNV)‐high (serous‐type) tumors than other racial groups. The most frequently mutated gene in Caucasian and Asian tumors was the *PTEN* gene unlike in black patients where it was the *TP53* gene. Both mutation and somatic copy number alteration revealed a significant amount of *TP53* mutations in black patients. It was also noted that there was a significantly higher frequency of somatic mutations in DNA mismatch repair genes in the tumors of Asian patients.^13^, ^14^ These molecular differences between racial groups likely also contribute to the varying disease response to treatment and to some of the disparity in health care outcomes. As such there may be a need for less discriminate genetic or molecular testing to determine risk and guide therapy among the racial/ethnic groups. Future research is needed, however, to explain the molecular differences in carcinogenesis and treatment responses within racial/ethnic groups.

The observation of a timely diagnosis and receipt of appropriate care are also important factors when assessing for healthcare disparities. In our review, we found that NH white women are more likely to present with early stage disease, undergo a hysterectomy, and have a lymphadenectomy performed (10 or more nodes), as compared to NH black women. There were, however, no significant differences found in the receipt of adjuvant therapies between these two groups. We also found that women, treated at a less than or equal to 2‐week time from diagnosis to intervention had a poorer outcome compared to their counterparts who received treatment between 2 and 4 weeks from diagnosis or later. Factors that may account for this difference include the presence of an aggressive/faster growing histologic diagnosis such as carcinosarcomas warranting sooner intervention, with an overall poor prognosis or more symptomatic patients with larger volume disease requiring earlier management. More late stage patients also received surgery in the less than 2‐week interval.

Our study is not without limitations, due to the retrospective nature of our study, decisions regarding surgery, type of adjuvant treatment, and timing were likely confounded by other factors. The registry may not completely capture all treatment data, especially treatment that is performed in the outpatient setting; however, in our cohort, treatment data were available for 95% of the population. In addition, the database does not include information pertaining to the specifics of provider level of training, expertise, treatment decisions, type of chemotherapy received, extent of residual disease after surgery, molecular profiling data, and completion rates of for example chemotherapy or radiation. Also provider factors including provider beliefs, biases, and expectations cannot be accounted for with a database study. The California Cancer Registry like other databases is subject to potential reporting errors although validation of data accuracy is carried out as best as is possible by internal monitoring and communication with reporting sites.

Despite the limitations, we think this study highlights the stark differences in prognosis and outcomes for NH black women diagnosed with high‐grade endometrial cancers. The drivers for worse outcomes among NH black patients may include limitations in access to care as well as intrinsic factors that are still unexplained. Given the steep rise in endometrial cancer rates, it is imperative to continue investigations into the main drivers of this racial gap in order to provide optimal cancer treatments for minority groups.

Increasing the awareness of patients and healthcare professionals of the signs and symptoms of uterine cancer and the differences in tumor type distribution among ethnic groups may also help reduce the survival difference by prompting earlier referral to subspecialty care. With the increased awareness and concern around ethnic/racial disparities in the care and outcomes of endometrial cancer patients, organizations such as Society of Gynecologic Oncologists (SGO) are championing efforts to eliminate disparities. There will be also the development of the Endometrial Cancer Action Network for African‐Americans (ECANA) to foster patient‐centered research in endometrial cancer disparities. The complex issue of healthcare disparity requires a multi‐pronged approach addressing patient, providers, and system factors.

## CONFLICT OF INTEREST

No conflict of interest from any of the listed authors.
